# Platelets, Thromboinflammation and Neurovascular Disease

**DOI:** 10.3389/fimmu.2022.843404

**Published:** 2022-03-04

**Authors:** Ying Sun, Harald F. Langer

**Affiliations:** ^1^ Cardioimmunology Group, Medical Clinic II, University Heart Center Lübeck, Lübeck, Germany; ^2^ University Hospital, Medical Clinic II, University Heart Center Lübeck, Lübeck, Germany; ^3^ DZHK (German Research Centre for Cardiovascular Research), Partner Site Hamburg/Lübeck/Kiel, Lübeck, Germany

**Keywords:** platelets, neuroinflammation, multiple sclerosis, Alzheimer`s disease, thrombosis, stroke

## Abstract

The brain and spinal cord are immune-privileged organs, but in the disease state protection mechanisms such as the blood brain barrier (BBB) are ineffective or overcome by pathological processes. In neuroinflammatory diseases, microglia cells and other resident immune cells contribute to local vascular inflammation and potentially a systemic inflammatory response taking place in parallel. Microglia cells interact with other cells impacting on the integrity of the BBB and propagate the inflammatory response through the release of inflammatory signals. Here, we discuss the activation and response mechanisms of innate and adaptive immune processes in response to neuroinflammation. Furthermore, the clinical importance of neuroinflammatory mediators and a potential translational relevance of involved mechanisms are addressed also with focus on non-classical immune cells including microglia cells or platelets. As illustrative examples, novel agents such as Anfibatide or Revacept, which result in reduced recruitment and activation of platelets, a subsequently blunted activation of the coagulation cascade and further inflammatory process, demonstrating that mechanisms of neuroinflammation and thrombosis are interconnected and should be further subject to in depth clinical and basic research.

## Introduction

The central nervous system (CNS), which includes the brain and spinal cord, is considered an immune privileged organ system. It contains a dense vascular network, and the vascular border forms a tight barrier, the so-called blood-brain barrier (BBB), regulating entry from outside the system. The BBB plays an important role in maintaining the separation of the CNS from the systemic immune system but the presence of the blood–brain barrier, does not, on its own, provide immune privilege (1).

Neurovascular diseases are disorders of the central nervous system, with its most common pathologies Alzheimer’s disease (AD), multiple sclerosis (MS) and ischemic stroke, which are all associated with abnormal neurovascular activation and thromboinflammation. This review focuses on describing how innate and adaptive immunity modulate the immune response in neuroinflammation, and we highlight the interaction of different (immune) cells in the CNS. During neurovascular inflammation resident immune cells such as microglia contribute to local inflammation, further immune cells such as effector T cells are recruited through the damaged blood-brain barrier with increased permeability and propagate the inflammatory reaction through of the release of inflammatory signals. Particularly in ischemic stroke, but also in other neurovascular diseases, inflammatory mediator infiltration causes platelet activation. Recent evidence indicates that beyond their role in forming a thrombus, platelets have both a promotional and modulating role in neuroinflammation. In the following, we discuss advances in understanding molecular mechanisms in neurovascular inflammation focusing also on previously neglected cells such as platelets, targeted neurovascular therapy and translation of experimental treatment strategies.

## Inflammation as a Central Principle in Neurovascular Disease

**Figure 1:**
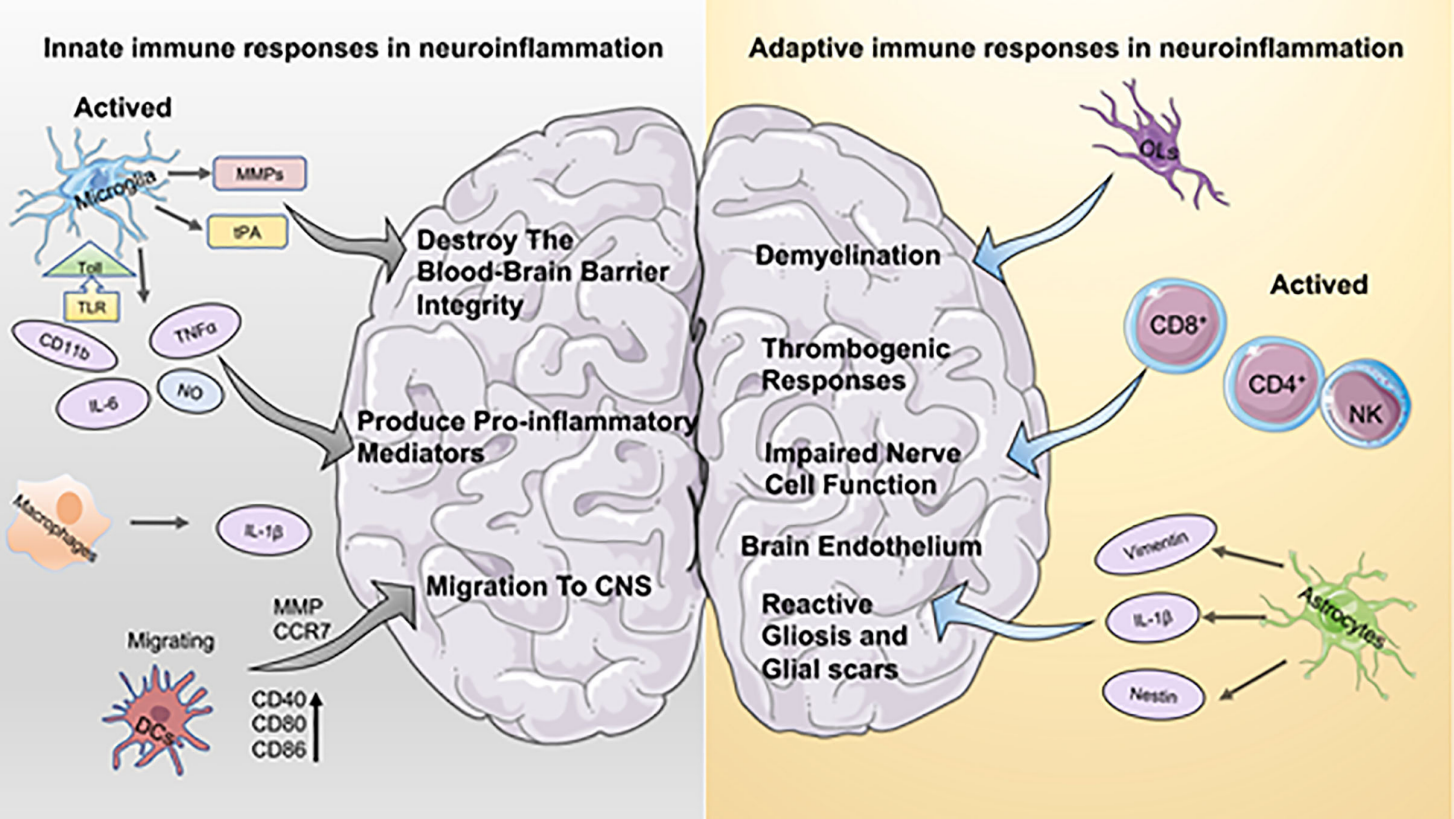
Aspects of innate immune and adaptive immune responses in neuroinflammation. On the left the innate immune response to neuroinflammation is depicted. Microglia hold a dominant role in innate immunity, and activated microglia contribute to both the disruption of the blood-brain barrier and the production of pro-inflammatory mediators. Macrophages secrete higher levels of IL-1b, which has a pro-inflammatory effect similar to that of microglia. DCs activate the immune response by interacting with T cells, which promote recruitment and migration of further immune cells to the CNS. The right part is adaptive immunity. Oligodendrocytes (OL) play a crucial role in inflammation-induced demyelinating diseases. Astrocytes are important for brain endothelium homeostasis *in vivo*. In addition, astrocytes contribute to the development of reactive gliosis and post-ischemic formation of neuroglial scarring at sites of local ischemia.

Inflammation is considered a key factor in disease development. In the cardiovascular system, dysregulated immune responses and oxidative stress play an essential role in diseases such as atherosclerosis with its severe late sequelae including myocardial infarction (2). In the central nervous system, neuroinflammation is now recognized as a decisive pathophysiological factor in diseases of the brain and/or spinal cord, and inflammation is clearly associated with neurodegenerative diseases and CNS injury (e.g., Ischemic Stroke, Multiple Sclerosis, Alzheimer’s disease, etc.). As the CNS is a particularly sensitive system with limited capacity for regeneration, the concept of immune activation with immediate, mid-term and long-term consequences for tissue remodeling within the CNS are thought to be critical for the course of many diseases. Particularly, the concept of CNS as an “immune-privileged” organ system has been subject to various investigations over the last decades. Current data indicate the presence of resident CNS macrophages known as microglia cells within the CNS, but there is also a wide body of evidence suggesting the active interaction of the CNS with peripheral immune cells (3). Data indicate that immune privilege within the CNS varies throughout the different compartments of the system, being most pronounced in the parenchyma tissue or “white matter” (1). Microglia mediate mechanisms of innate immunity in the CNS by producing cytokines and chemokines, and by the propagation of inflammatory intracellular signals. Recent studies have shown that platelets are also a potential branch of innate immunity that interact with other cells involved in the immune response to the CNS (4). When CNS lesions occur, along with resident immune cells of the CNS such as microglia, platelets are activated, initiate adaptive immunity, promote the recruitment of peripheral cells to the brain, and increase the permeability of the BBB (**Figure 1**).

### Innate Immune‐Mediated Responses Following Neuroinflammation

Generally, in normal (uninjured) tissue, antigens are taken up by antigen presenting cells (dendritic cells, DCs), and subsequently transported to the lymph nodes. Alternatively, soluble antigens can drain into the lymph nodes. However, in the CNS, DCs are not thought to be present in normal parenchymal tissue or perivascular space although they are present in the meninges and choroids plexus (1). DCs are antigen-presenting cells and mature DCs activate the immune response by interacting with T cells, whereby they provide a connection between innate and adaptive immunity (2). Thus, the CNS is thought to be limited in its capacity to deliver antigens to local lymph nodes and cause T-cell activation (5). DCs are sparsely found in healthy brain tissue, mostly in vascular-rich areas such as the meninges, choroid plexus and spinal cord where subpopulations of CNS-resident DCs are observed (6). In the peripheral blood and cerebrospinal fluid (CSF) of neuroinflammatory patients, activated DCs recruit and accumulate, signaling their active involvement in immuno-pathogenesis (7, 8). DCs migration across endothelial cell monolayers is partially dependent on matrix metalloproteinases (MMP), and migrating DCs express higher levels of CD40, CD80 and CD86, which co-stimulate molecules and induce T cell proliferation (9). The chemokine CCR7 has been shown to be associated with the migration of DCs (10), and both CCL19 and CCL21 as ligands for CCR7 have been identified on DCs in CNS lesions, supporting the idea that DCs recruited during CNS inflammation retain their ability to migrate to the periphery with CNS (auto) antigens and activate T cells (6, 10).

The CNS defensive wall against intruders is the blood-brain barrier (BBB) (11). When the BBB is dysfunctional, a transient innate immune response is immediately activated. Microglia are the resident macrophages of the CNS, which represent 5–20% of the glial population (4). The grey matter generally contains more microglial cells than the white matter (12). Microglia cells have important physiological functions in maintaining tissue homeostasis, including the promotion of neuronal survival and mediating synaptic plasticity (13, 14). On the other hand, they may also contribute to CNS pathologies. At rest, microglia are described as small cells with broad prominent branches (15). When microglia are activated, their morphology and function are altered, and during systemic inflammation microglia cells function similarly to macrophages, release cytokines, secrete extracellular matrix metalloproteinases (MMPs) and tissue-type plasminogen activator (tPA), which can destroy the BBB and increase its permeability (16). The neuroinflammatory response is mediated by several key proinflammatory cytokines (IL-1β, IL-6, and TNF), chemokines (CCL2, CCL5, CXCL1), secondary messengers (NO and prostaglandins), and reactive oxygen species (ROS) (17, 18). This activation state of microglia can be sustained for several weeks. Similar to macrophages, microglia cells express toll-like receptors, respond to TLR ligands and produce pro-inflammatory mediators (12). Sun et al. found that Isoflurane preconditioning (IP) provided neuroprotection and inhibited microglia activation by directly regulating TLR4 expression and 2% IP attenuated neurological deficits, reduced infarct volume, attenuated apoptosis and decreased microglia activation in the ischemic penumbra (19). Furthermore, it was reported that homocysteine enhanced MCAO-induced microglial cell activation and inflammatory cytokine secretion, including TNF and IL-6 production (20). AG490, one of the inhibitors of the JAK pathway, decreased homocysteine-enhanced STAT3 phosphorylation and significantly reduced homocysteine-induced microglia activation and production of TNF or IL-6, suggesting that homocysteine may trigger brain injury through the JAK2/STAT3 signaling pathway (20).

Another type of innate immune cell type involved in neuroinflammation are macrophages. The role of macrophages and microglia cells in ischemic stroke is complementary in many ways and, therefore both cells are often addressed in the same context. However, microglia and macrophages produce different types of major inflammatory factors: microglia produce relatively high levels of reactive oxygen species (ROS) and TNF in contrast to macrophages, which produce higher levels of IL-1β (21). Another study demonstrated that microglia, which are essential for neuroprotection, gradually switched from their neuroprotective and anti-inflammatory phenotype to an inflammatory phenotype with age (22). The number of CD11b^+^ CD45^low^ CXCR2^+^ and CD11b^+^ CD45^low^ CCR1^+^ microglia cells was increased compared to young mice, and these changes were associated with evidence of increased microglia activation, including increased expression of CD11b, MHCII, CD68, TNF, and IL-6. Several studies have indicated that microglia aggravate secondary inflammation-related injury during neuroinflammation, that microglia are known as pro-inflammatory reactors and enhance post-stroke injury, increase cerebrovascular permeability, and contribute to neuronal injury and apoptosis (16, 23–25). It is interesting to note that the effect of microglia on neuroinflammation is two-sided (26). Trigger receptor 2 (TREM2) expressed on myeloid cells is an important innate immune receptor in the brain, mostly localized on microglia cells, and was described to mediate the phagocytosis of damaged brain cells (27). TREM2 gene silencing exacerbates the inflammatory response, increases neuronal apoptosis and infarct volume, and further exacerbates neurological dysfunction (28). Interestingly, TREM2 knockout mice have poor neurological recovery with decreased viable brain tissue in the ipsilateral hemisphere (28). TREM2 has furthermore been implicated in protection against cerebral ischemia/reperfusion injury through post-ischemic inflammatory responses and neuronal apoptosis (27). Another important mediator in neuroinflammation is ST2, a member of the interleukin (IL) 1 receptor family. Its ligand IL-33 activates microglia to release IL-10 *in vitro*, which is crucial for its neuroprotective effects. ST2 deficiency shifts microglia/macrophages to an M1-like phenotype, which expands cerebral infarction and exacerbates long-term behavioral deficits after ischemic stroke (29).

Besides “classical” innate immune cells, platelets have been found to be linked to inflammation and are emerging as a potential effector cell type of the inflammatory neurovascular response (4, 30). Activation of DCs, B cells, and T cells can be mediated by platelet expression of CD40L, suggesting that platelets provide a cross-link between innate and adaptive immunity. Neutrophils are a crucial component of the innate immune response, killing pathogens through phagocytosis, protease degranulation and release of reactive oxygen/nitrogen species (ROS and RNS) (31). A minority of neutrophils are present in the meninges, pia membranes and cerebrospinal fluid rather than in the brain parenchyma, and neutrophils need to rely on other resident cell secretory mediators to induce recruitment of neutrophil (32, 33). For example, in animal models of ischemic stroke, injury induces the release of DAMP from damaged cells, which activates resident cells to produce chemokines CXCL2 and CXCL8, leading to neutrophil recruitment to the CNS (33–35).

### Adaptive Immune‐Mediated Responses Following Neuroinflammation

The activation of adaptive immunity in the CNS is complex and differs from the rest of the body because of the presence of the BBB, the high density of blood vessels, the lack of “classical” antigen-presenting cells and of a “classical” lymphatic system. Although there is no conventional lymphatic system in the CNS, the drainage of antigens from CNS tissue into the cervical lymph nodes has been demonstrated. The response elicited in the lymph nodes to CNS antigens is skewed towards B-cells. DCs from cerebrospinal fluid have been found to migrate to B-cell follicles of cervical lymph nodes (36).

The skewing of the response to antigen from the CNS towards a humoral response means that a more dangerous inflammatory T-cell response can be avoided. The induction of systemic tolerance to an antigen introduced into the CNS has been previously shown (37).

This was seen in the absence of the T-cell mediated inflammatory “delayed type hypersensitivity reaction” (DTH) when the antigen was reintroduced in another part of the body, which is analogous to Anterior Chamber-Associated Immune Deviation (ACAID) in the eye.

During ischemic stroke, it has been established that T lymphocytes play a major role for pathophysiology and recovery from injury (38–40). Within hours after tMCAO (an established wire induced *in vivo* model for ischemic stroke featuring ischemia-reperfusion injury), T lymphocytes are recruited in large numbers around the borders of the infarcted region (41). In particular, cytotoxic CD8^+^ lymphocytes have been detected in the ischemic brain within 3 hours after ischemic stroke, whereas CD4^+^ T cells and natural killer cells are recruited during the first 24 hours and peak at 72 hours after reperfusion (12, 16, 26). At 3 days and 1 month after ischemic stroke, peripheral T cell infiltration was significantly increased in the ischemic hemisphere compared to the contralateral hemisphere (42, 43). CD4^+^ and CD8^+^ T cells contribute to the inflammatory and thrombogenic responses, brain damage, and neurological deficits associated with experimental ischemic stroke. Strikingly, in T lymphocyte-deficient mouse models, a significant reduction in infarct volume and improvement in neurological deficits have been measured (43, 44).

Together, when adaptive immune T cells are activated in the CNS, the release of immune mediators promotes a widespread local inflammatory response that increases the permeability of the BBB, thereby eliminating the transport limitations and barrier function of the BBB. Extensive CNS infiltration by inflammatory cells and entry of plasma proteins eventually leads to demyelination, edema, impaired nerve cell function, and neurobehavioral impairment (45).

### Other Cells Involved in Neuroinflammation

Astrocytes contribute to the physiological homeostasis and pathological dysregulation of the CNS (46). They help to maintain the molecular homeostasis between nervous and immune by synthesizing glycogen and providing energy substrates for neurons, transporting major ions and protons, removing and disintegrating neurotransmitters, and releasing neurotransmitter precursors and reactive oxygen scavengers (47–49). Astrocytes form a key structural component of the BBB, as they stretch and wrap their end-feet around the cerebral vascular system. The astrocyte end-feet are directly in contact with endothelial cells *via* the channel-forming protein water channel protein 4, Kir4.1, and the gap junction-forming protein Connexin 43, allowing diffusion of water, ions, and soluble factors (50, 51). Astrocytes regulate properties of the BBB of the adult mouse brain endothelium *in vivo* through specific BMP signaling mechanisms on the astrocytic end-feet. Accordingly, damage to astrocytes leads to a gap in the BBB (46, 52). In multiple sclerosis (MS) tissue, degradation with endothelial ligand proteins (e.g., OCLN, VE-calmodulin) is detected with severe astrocyte proliferation around the blood vessels in the center of active lesions and an increased number of GFAP ^+^ end-feet (50, 53). These proteins are essential for the normal function of the BBB. Astrocytes respond to CNS disease and trauma through cell proliferation, increased branching and increased cell size, a phenomenon often defined as astrocyte hyperplasia, which has both positive and negative effects on neurological outcomes (54). Following local ischemia, astrocytes release wave proteins, nestin, and IL-1β to form a dense barrier, which contributes to the development of reactive gliosis and the post-ischemic formation of glial scars (16, 55, 56). Upregulation of intermediate filament glial fibrillary acidic protein (GFAP) as a commonly used marker to identify astrocyte hyperplasia can be correlated with the reactive/astrocytic phenotype acquired by astrocytes during CNS stress and disease (57). In Alzheimer’s patients, similar to microglia, astrocytes release cytokines, interleukins, nitric oxide and other potentially cytotoxic molecules when exposed to soluble amyloid (Aβ), thereby exacerbating the neuroinflammatory response. Furthermore, reactive astrocytes play an essential role in this disease (58). Similar to microglia, astrocytes are located in the plaques surrounding soluble amyloid (Aβ) deposits and release cytokines, interleukins, nitric oxide and other potentially cytotoxic molecules that exacerbate the neuroinflammatory response. GFAP levels determine the intensity of proliferation of reactive astrocytes (46).

Oligodendrocytes (OLs) are the myelinating cells of the CNS, which form a functional unit with axons and play a vital role in axonal integrity. OLs are vulnerable in the acute phase of ischemia and form myelin sheaths on sprouting axons in the chronic phase. Mature oligodendrocytes form myelin sheaths that are used to sprout axons in ischemic brain tissue (42, 59). Ischemic hypoxia caused by excessive activation of glutamate and ATP receptors, oxidative stress and disruption of mitochondrial function all cause severe damage to OLs (60, 61). They bind to their respective receptors on the plasma membrane of OLs and lead to an influx of Ca^2+^ ions, which under physiological conditions act as chemical signals to stimulate OL differentiation and myelin formation oligodendrocyte production is the main brain repair process after ischemic stroke (62). Because OLs are incapable of self-renewal, oligodendrocytes are dependent on oligodendrocyte progenitor cells (OPCs) in the corpus callosum, striatum and subventricular zone (63). Ischemic stroke induces OPC proliferation and migration, which contributes to the production of mature oligodendrocytes and thus to neuronal recovery (64). Investigations have indicated that inflammatory cytokines have a key role in demyelinating diseases. Tumor necrosis factor-α delays myelin formation, induces oligodendrocyte apoptosis, and inhibits OPC proliferation and differentiation, while interferon-γ (IFN-γ) also has a concentration-dependent effect on oligodendrocyte lineages and induces oligodendrocyte apoptosis and reduces OPC proliferation (65–67). Transplantation of bone marrow mesenchymal cells was detected to significantly increase the number of oligodendrocyte progenitors in the ischemic hemisphere, as well as the number of mature oligodendrocytes in the ischemic border adjacent to the myelinated axons (59, 68). The investigation of oligodendrocytes could be a target for the improvement of function after stroke.

### Neuroinflammation in Diseases of the Central Nervous System

Neuroinflammation promotes dysfunctional neuron-microglia-astrocyte crosstalk and is a common feature of many neurodegenerative diseases. It maintains microglia cells in a detrimental reactive state, thereby exacerbating neuronal damage (69).

Alzheimer’s disease is the most common type of dementia and is induced by extracellular β-amyloid deposition and abnormal tau phosphorylation (70). The immunostimulatory molecule lipopolysaccharide (LPS) is used to trigger systemic inflammation in the majority of animal models (71). Elevated levels of peripheral systemic LPS or peripheral pro-inflammatory cytokines result in transient neuronal death, and systemic LPS exposure does result in increased deposition of Aβ 1-42 and phosphorylated tau (p-tau) levels in the wild-type rodent brain; thus these pathological changes directly or indirectly induce synaptic and neuronal dysfunction and ultimately lead to clinical dementia (70, 72). In addition, Helicobacter pylori (H. pylori) infection has been shown to be associated with the development of AD. Intraperitoneal injection of H. pylori TN2GF4 filtrate resulted in elevated Aβ levels and induced spatial learning and cognitive impairment through interrupting the synaptic function in wild-type rats (73).Furthermore, H. pylori TN2GF4 infection induced significant tau hyperphosphorylation (74). These immune factors circulate in the blood and eventually affect neuroinflammation through neural and humoral pathways. The binding of Aβ to microglia-expressed receptors CD36 or TLR4 leads to the production of inflammatory cytokines and chemokines *in vitro*. In studies in transgenic mouse models of AD, the release of TNF from microglia in response to Aβ is triggered by the interaction of CD40 with CD40L or by the involvement of TLR4 (75). Recent studies have found higher levels of inflammatory cytokines and neuronal/glial markers in the cerebrospinal fluid of patients with acute neuritis compared to controls, with no differences in amyloid-related markers (76). Together, the above studies suggest an association between neuroinflammation and AD.

Multiple sclerosis (MS) is an immune-mediated disease of the central nervous system that leads to demyelination and chronic neurodegeneration. Over-activation of T cells and microglia is a hallmark of the pathogenesis of human MS (77). Most people with MS suffer from progressive disability. MCP-1/CCL2, IL-8 and MIP-1α/CCL3 have been demonstrated to be important pro-inflammatory mediators involved in the pathogenesis of experimental autoimmune encephalomyelitis (EAE) and MS. IL-8 levels in the cerebrospinal fluid of MS patients were elevated compared to controls and correlated with the course of MS (78). Interestingly, activated microglia and macrophages in active MS lesions are a major source of oxidative stress. The HLA class II alleles DRB1*1501, DRB1*0301 and DRB1*1303, expressed on cells of the innate immune system, were identified with an increased risk of developing MS, while the HLA class I allele A2 was associated with a decreased risk (79, 80). During the adaptive immune phase of multiple sclerosis, CD4^+^ T cells enter the perivascular space and release cytokines that can affect astrocyte end-feet, leading to disturbance of oligodendrocyte and astrocyte homeostasis. The released inflammatory mediators open the blood-brain barrier and attract infiltration of monocytes and other lymphocytes (79). Reactive astrocyte-derived secreted frizzled-related protein 1 (SFRP1) promotes the upregulation of components of the hypoxia-induced factor-dependent inflammatory pathway and slightly regulates the downstream component of nuclear factor-κB in microglia. This increases the number of activated microglia and promotes HIF expression, however, the continued presence of SFRP1 is detrimental as it maintains a chronic inflammatory state (69). In the peripheral immune system, mRNA for the neuromodulator TAAR1, which is present in whole peripheral blood mononuclear cells, activates and upregulates mRNA levels of pro-inflammatory cytokines, including IL-6, IL-1β and TNF. Under standard conditions, resting macrophages located on TAAR1 exhibit protein expression that is predominantly localized to the nucleus, in contrast to the intracellular translocation shown during pro-inflammatory stimulation (81, 82). Jing et al. found that IFP35 family proteins act as pro-inflammatory molecules that activate macrophages, stimulate DCs and promote the differentiation of naive T cells to Th1 and Th17 cells, making them a new target for studying the pathogenesis of MS (77).

Recent insights move neuroinflammation into the center of interest after ischemic stroke, but also how inflammation induces stroke onset. The neuroinflammatory response triggers and persists after ischemic stroke injury in multiple aspects, including necrotic cells, debris, and ROS. after ischemic injury, endothelial damage leads to impaired nitric oxide production, which exacerbates oxidative stress, leading to activation of proinflammatory genes and inflammatory cells (83). Furthermore, microglia and astrocytes are activated. The exact mechanisms, how the different cell types interact and their contributions are separately described in the previous sections.

## Thrombosis as a Central Principle in Neurovascular Disease

Hemostasis is a protective process that seals damaged vessels and ultimately supports vascular recovery through limited production of thrombin and fibrin. Pathologic thrombosis occurs in veins or arteries when abnormal clotting is found at the site of injury or inflammation (84). Inflammation and coagulation interact extensively in the pathogenesis of various vascular diseases, with inflammation being an emergency response to the entry of infectious agents into the vascular system. Inflammation leads to activation of coagulation, while coagulation vice versa significantly affects inflammatory activity (85). Procoagulant substances expressed by inflammatory cells may initiate coagulation activation, such as the migration of small amounts of thrombin (II) and factor IXa (IXa) from the tissue factor surface to the platelet surface, causing activation of factors V, VIII, XI and platelets (86). Subsequently, on the platelet surface, activated factors aggregate and generate thrombin, all of which lead to platelet-fibrin thrombus formation (84). Cerebrovascular diseases can trigger stroke and lead to other forms of neurological dysfunction and vascular degeneration. Ischemic strokes are primarily the result of a carotid or a cerebrovascular disease (87). Approximately 80% of strokes are caused by focal cerebral ischemia due to arterial embolism. In approximately one-third of patients with ischemic stroke, the cerebral embolism originates in the heart, particularly caused by atrial fibrillation (88). Therefore, coronary artery and pathological thrombus formation in myocardial infarction and stroke are considered to have partially overlapping molecular pathways (89). Chemokines are key players in atherosclerosis, such as macrophage migration inhibitory factor (MIF), which regulates upstream signaling of host innate and adaptive immune responses (90–92). While MIF is dysregulated, leukocytes are recruited to trigger foam cell formation and advanced plaque remodeling in atherosclerotic vascular inflammation (93). MIF inhibitors were developed by interacting with CXCR4 extracellular domain-derived peptides on the surface of MIF. CXCR4 mimics act as soluble chemokine receptors to specifically target and block atherogenic inflammation (94).

Studies in cerebral venous thrombosis (CVT) have shown extensive crosstalk between inflammatory cytokines such as C-reactive protein, IL-6 (interleukin-6), etc. and coagulation factors, demonstrating that inflammation plays an important role in thromboembolic disease (95). Expression of procoagulant substances by inflammatory cells in unstable plaques (especially tissue factor) may initiate coagulation activation by binding factor VIIa, which produces thrombin (factor IIa) that activates platelets and leads to platelet-fibrin thrombosis (85). It is notable that inflammation at admission in patients with CVT is positively correlated with cerebral venous thrombosis severity and prognosis (95). Thus, an intricate mutual interrelationship between inflammation and thrombosis exists in diseases of the central nervous system.

## The Contribution of Platelets to Both Thrombosis and Inflammation in Neurovascular Disease

**Figure 2:**
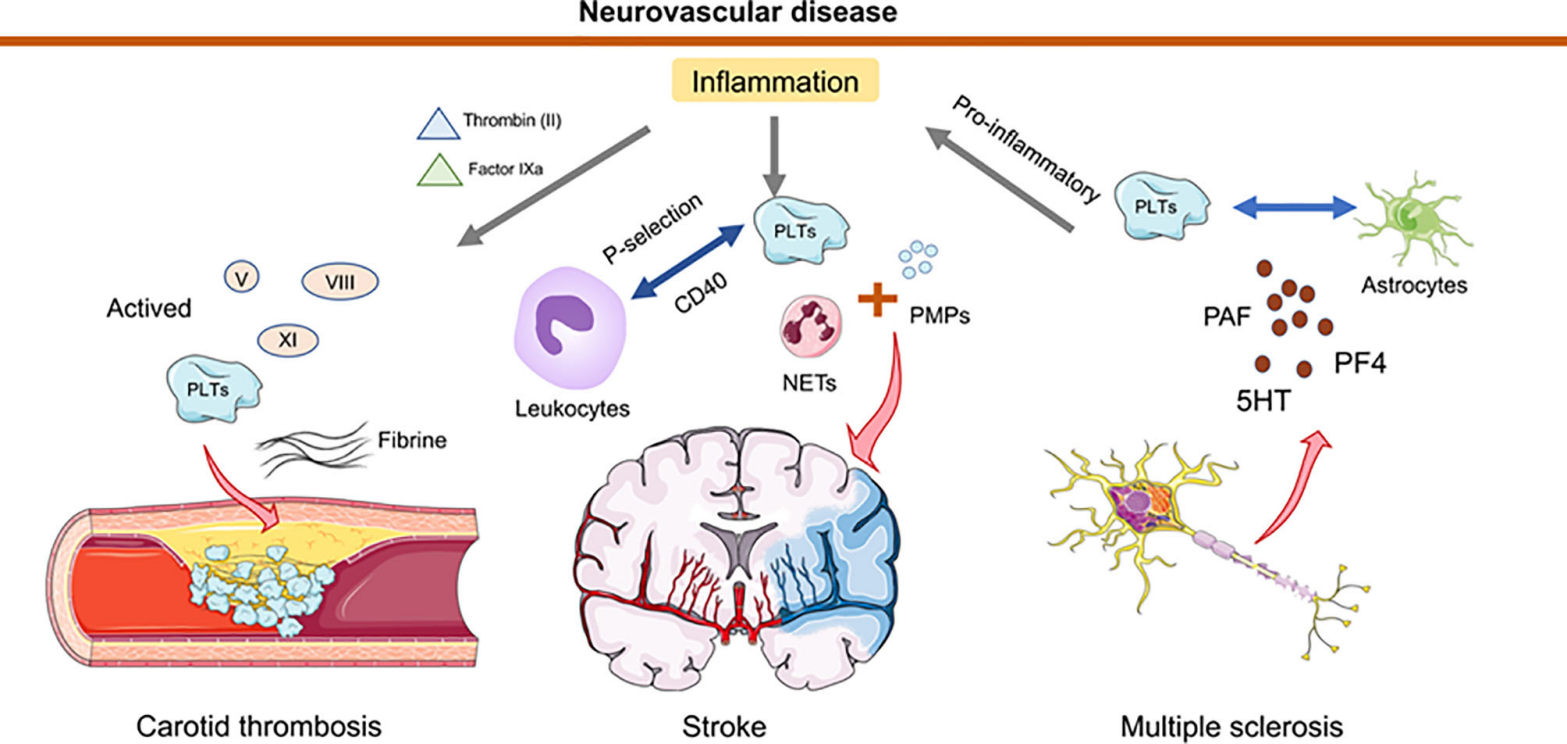
Schematic representation of how platelets and thrombosis can affect neurovascular disease. In the inflammatory state, thrombin (II) and factor IXa are transferred from tissue factor to the platelet surface, leading to activation of factors V, VIII, XI. Collective activation of these factors during stroke trigger carotid thrombosis. Neutrophil extracellular traps (NETs) activated by platelets (PLTs), and PLT-derived granules (PMPs) have been detected in elevated amounts in the plasma of stroke patients. Platelets interact directly with circulating leukocytes by altering the surface expression of P-selectin or CD40. In multiple sclerosis, platelets degranulate upon interaction with astrocytes and release PAF, PF4 and 5HT. This degranulation triggers T cell proliferation and pro-inflammatory cytokine production. Furthermore, platelets mediate the recruitment of leukocytes to and the activation of local innate immune cells at the site of inflammation and contribute to tissue damage.

Recent studies have revealed that platelets provide an important contribution to the functionality of the immune system and interact intensively with other immune cells (including neutrophils, macrophages, DCs) (33, 96, 97). In neuroinflammation, the interaction of platelets with immune cells that may promote/inhibit the inflammatory is currently subject to intense research (96, 98, 99). Platelet interactions during thrombosis are strongly mediated and stabilized by the platelet fibrinogen receptor GP2b/3a (100). By analyzing the levels of neutrophil extracellular traps (NETs), activated platelets (PLTs), PLT-derived particles (PMPs), and exposed phosphatidylserine (PS) detected in the plasma of 55 stroke patients and 35 healthy controls, Zhou et al. demonstrated that NETs bearing PS, activated PLTs, and PMPs accumulate locally at the site of carotid thrombosis, and that NETs with functional PS induce thrombin generation and PLT activation, leading to an increased risk of atherosclerotic thrombosis (101). Another study has illustrated that AnxA1 provides protection by modifying the platelet phenotype from pathogenic to regulatory in cerebral ischemia/reperfusion (I/RI) and by affecting integrin (α _IIb_ β _3_) activation to reduce the propensity of platelets to aggregate and cause thrombosis (102). In response to infectious conditions or inflammatory stimuli, platelets interact directly with circulating leukocytes by altering the surface expression of P-selectin or CD40 (103). Because activated platelets secrete chemokines, cytokines, and other pro-inflammatory mediators, they can trigger and modulate inflammation at the site of injury. In the MS mouse model, platelets degranulate upon interaction with other cells, such as astrocytes, and release PAF, PF4, and 5HT, components of platelet granules (98). Degranulation and subsequent release of several platelet-derived soluble factors contribute to CD4 T-cell proliferation and pro-inflammatory cytokine production. By contrast, the formation of platelet aggregates leads to reduced T cell proliferation and pro-inflammatory cytokine production. In addition, degranulated platelets activate endothelial cell mediators that promote the migration of CD4^+^ T cells into the central nervous system (98). In a mouse model of pentylenetetrazole (PTZ)-induced epilepsy, platelet-neuronal glycolipid interactions enhanced epileptic seizures (104). Therapeutic blockade of platelet GPIb α may limit the deleterious effects of excessive inflammation while minimizing the bleeding complications of thrombocytopenia in the brain (33, 105). When the blood-brain barrier is disrupted after brain injury, platelet-neuron synapse-like structures are generated, releasing 5-HT and leading to enhanced neuronal plasticity and upregulation of PSD95 formation in dendritic spines (106). A significant reduction in tissue apoptosis after PLT depletion was observed in both middle cerebral artery embolization models (107) (**Figure 2**).

## Translational Approaches to Target Thromboinflammation in (Neuro-) Vascular Disease

In targeted neurovascular therapy, the prevention of thrombosis and the reduction of excessive immune responses to vascular or tissue injury are two crucial concerns. Thrombolytic therapy with tissue fibrinogen activator (tPA) in acute stroke increases the risk of cerebral hemorrhagic transformation and angioedema. A significant reduction in tPA-induced intracerebral hemorrhage, edema, and infarct volume was observed in plasma prokinetic peptide-releasing enzyme-deficient (Klkb1) mice (108). Early blockade of platelet tethering and activation by GPIb or GPVI is an important checkpoint for thrombogenic and proinflammatory pathways in ischemic stroke. However, blockade of GPIb or GPVI has no effect on hemostasis in acute experimental stroke (97). Aspirin, one of the earliest drugs applied to prevent thrombosis, targets COX-1 in platelets to block prostaglandin production from arachidonic acid. A more selective 12-LOX inhibitor, ML355, was described and demonstrated effective inhibition of platelet activation *in vivo* (109). A novel snake venom-derived GPIb antagonist, Anfibatide, showed dramatically improved ischemic lesions by way of dose modulation. Compared to untreated MCAO mice, these mice had smaller infarct volumes and reduced the amount of GPIbα, vWF, and fibrin(pro) accumulation in the ischemic hemispheric vascular system (110, 111). Unfortunately, neither immunoreactive cytostatic agents nor inflammatory mediator reduction have yielded good results in the immune response after ischemic stroke. In recent years an increasing number of clinical trials of mesenchymal stem cells (MSCs) transplantation in patients after ischemic stroke have shown that MSCs do not cause significant side effects, and positive results of cell transplantation have been observed in some trials (112, 113).

Antiplatelet therapy is effective for secondary prevention after an ischemic stroke or transient ischemic attack (TIA) (114). Even dual antiplatelet therapy has been applied in clinical trials (115). However, studies have reported that antiplatelet therapy increases the risk of bleeding and is positively associated with duration of therapy (116). Early and short-term dual antiplatelet therapy for minor stroke or high-risk transient ischemic attack (TIA) has been suggested by the clinical guidelines (116). In patients undergoing percutaneous coronary intervention (PCI) for acute coronary syndrome (ACS), dual antiplatelet therapy is used to prevent ischemic events in the peri- and post-procedure period (117). In contrast to the concept of a broad and strong anti-platelet therapy approach, recent studies have suggested more targeted therapies in order not to increase the risk of systemic bleeding. One example is to inhibit the platelet collagen receptor, thus aiming at exposed collagen at sites of atherosclerotic lesions, without significant impact on platelet mediated hemostasis. For instance, the novel drug Revacept inhibits platelet recruitment and activation through competitive inhibition with glycoprotein VI (GPVI). While this concept could be verified in a phase III trial, the net clinical benefit for the patients was not obvious, indicating that further investigations are warranted (116). The trial observed a lower risk of stroke in the colchicine group than in the placebo group (118). A further interesting approach is to treat inflammation in cardiovascular atherosclerotic diseases with colchicine. Colchicine interferes with many functions of leukocytes, including migration and degranulation (119, 120). Furthermore, this drug may work through its effects on cell adhesion molecules and inflammatory chemokines, such as interfering with the assembly of multimeric NLR family pyrin domain containing 3 (NLRP3) inflammatory vesicles. However, gastrointestinal side effects of colchicine are more frequent in the treatment group (i.e., nausea and flatulence) and should be used with caution for example in patients with comorbid gastrointestinal disorders (121).

In conclusion, thromboinflammation is clearly associated with neurovascular diseases and it contributes significantly to disease progression and tissue remodelling. Besides classical immune cells involved in disease pathology and tissue damage such as T cell, other inflammatory cells including microglia, platelets, and their sequelae thrombosis and inflammation play an important role in neurovascular disease. Investigating these cell types and associated mechanisms will help to bring up new molecular targets to approach these devastating and challenging diseases. Furthermore, targeted antithrombotic therapeutic strategies to treat the detrimental inflammatory response without increasing the risk for bleeding could be an effective adjunctive therapy to reduce the severity and brain damage in multiple sclerosis stroke or other neurovascular diseases.

## Author Contributions

YS wrote the manuscript in consultation with HFL. HFL conceptualized and submitted the manuscript. All authors contributed to the article and approved the submitted version.

## Funding

This work was supported by the Volkswagen Foundation (Lichtenberg program), the DZHK (German Research Centre for Cardiovascular Research), partner site Hamburg/Lübeck/Kiel (STO Projekt F280404) and an EU Grant (ERAPERMED2020-245 PROGRESS “PRecisiOn medicine in CAD patients”) to HFL.

## Conflict of Interest

The authors declare that the research was conducted in the absence of any commercial or financial relationships that could be construed as a potential conflict of interest.

## Publisher’s Note

All claims expressed in this article are solely those of the authors and do not necessarily represent those of their affiliated organizations, or those of the publisher, the editors and the reviewers. Any product that may be evaluated in this article, or claim that may be made by its manufacturer, is not guaranteed or endorsed by the publisher.
